# Editorial: Multi-Scale Computational Cardiology

**DOI:** 10.3389/fphys.2022.847118

**Published:** 2022-02-07

**Authors:** Ling Xia, Henggui Zhang, Dingchang Zheng

**Affiliations:** ^1^Department of Biomedical Engineering, Zhejiang University, Hangzhou, China; ^2^Biological Physics Group, The University of Manchester, Manchester, United Kingdom; ^3^Research Centre of Intelligent Healthcare, Coventry University, Coventry, United Kingdom

**Keywords:** computational cardiology, computational fluid dynamics - CFD, cardiac electrophysiology, modeling and simulation, ECG signal processing

Computational cardiology plays an increasingly important role in elucidating the physiological and pathological mechanism(s) of normal and abnormal cardiac functions, guiding the development of new diagnostic technologies and treatment strategies, and predicting treatment outcomes. Recently, we called for paper submissions on the Research Topic of *Multi-Scale Computational Cardiology*. Twelve manuscripts were finally accepted for publications, where important scientific progress has been reported, covering computational fluid dynamics of circulation system (four papers), multi-scale cardiac electrophysiology modeling and simulation (three papers), and AI-related ECG signal processing (five papers). There are briefly summarized below.

Coronary artery disease (CAD) is the leading cause of death globally, but there is a lack of studies on the three-dimensional (3D) geometric characteristics of coronary plaque. Liu, Wingert et al. investigated whether coronary plaques of different sizes were consistent in geometric properties. Nineteen cases with symptomatic stenosis caused by atherosclerotic plaques in the left coronary artery were included in their study. 3D coronary atherosclerotic plaques and calcifications were identified, reconstructed, and manually revised based on computed tomography angiography images. Multidimensional geometric parameters were measured on the 3D models of plaques and calcifications. Linear and non-linear (power function) fittings were used to investigate the relationship between multidimensional geometric parameters. Their results disclosed the geometric consistency among coronary atherosclerotic plaques of different sizes.

Newtonian fluid model has been commonly applied in simulating cerebral blood flow in intracranial atherosclerotic stenosis (ICAS) cases using computational fluid dynamics (CFD) modeling, while blood is a shear-thinning non-Newtonian fluid. Liu, Lan et al. investigated the difference of cerebral hemodynamic metrics in CFD models with both Newtonian and non-Newtonian fluid assumptions, in patients with ICAS. The results suggested that Newtonian fluid model could be applicable for pressure ratio calculation, but caution needs to be taken when using the Newtonian assumption in simulating wall shear stress especially in severe ICAS cases considering the observable differences of wall shear stress between Newtonian and non-Newtonian estimations in areas with low shear rates distal to a stenosis.

It has been reported that 250,000 heart valve surgeries are performed worldwide each year. Among these, aortic valve (AV) diseases have become the second-leading cause of cardiovascular diseases due to their high morbidity and mortality. Numerical simulations of the AV dynamics can be applied to assess the hemodynamic performance, predict the effectiveness and persistence of surgical treatments, thereby guide AV disease management. Cai et al. presented a simulation study of different constitutive laws and fiber architectures for the AV on fluid-structure interaction. The results suggested that the strain energy function with exponential terms for both the fiber and cross-fiber directions could be more suitable for describing the AV leaflet mechanical behaviors.

Blood perfusion is an important index for the function of the cardiovascular system. It can be indicated by the blood flow distribution in the vascular tree. Li et al. proposed a novel fractal method for characterizing the distribution of blood flow in multi-scale vascular tree. Their validation on real arterial trees verified the ability of the produced parameters (fractal dimension and multifractal spectrum) in distinguishing the blood flow distribution under different physiological states. The results suggested that both the vascular structure and the blood flow distribution affect the fractal parameters for blood flow.

In recent years, ventricular tachycardia ablation strategy based on personalized virtual-heart technology has been researched and gradually applied to clinical practice. Due to the high demanding of computational resource of modeling, the arrhythmias induced in the models are usually simulated only for a few seconds. In clinic, however, it is common that arrhythmias last for more than several minutes and the morphologies of reentries are not always stable. So scientific evidence is required to demonstrate how long the simulation of arrythmias is efficient to match the arrhythmias detected in real patients. Tong et al.'s investigation suggested that 10 s simulation was sufficient to make arrhythmias simulation results stable.

Recent experimental evidence has indicated that mitochondrial depolarization promotes arrhythmogenic delays afterdepolarizations (DADs) in cardiac myocytes. However, the non-linear interactions among the Ca^2+^ signaling pathways, ROS, and oxidized Ca^2+^ /calmodulin-dependent protein kinase II (CaMKII) pathways make it difficult to reveal the mechanisms. Based on a recently developed spatiotemporal ventricular myocyte computer model, Pandey et al. concluded that the direct redox effect of ROS on ryanodine receptors (RyRs) plays a critical role in promoting Ca^2+^ waves and DADs under the acute effect of mitochondrial depolarization.

Computational modeling of the failing heart provides insights into mechanisms of arrhythmogenesis, risk stratification of patients, and clinical treatment. Sankarankutty et al. provided a microscopy-based foundation for modeling conduction in HF tissues. Their result suggested that conduction differs in the two etiologies due to the characteristics of fibrosis, highlighting the importance of the etiology-specific modeling of HF tissues and integration of medical history into electrophysiology models for personalized risk stratification and treatment planning.

Pace mapping is commonly used to locate the origin of ventricular arrhythmias, especially premature ventricular contraction (PVC). He et al. proposed a novel model based on spatial and morphological domains to predict the origin of PVC. The results showed that the proposed model was slightly superior to other models by achieving the most hits, the smallest estimated errors, and the biggest reduced distances for the PVC origin site estimation.

Remote ECG diagnosis has been gradually used in the clinical ECG workflow, especially for patients with pacemaker. An automatic detection pacing ECG method can help cardiologists reduce the workload and the rates of misdiagnosis. Ge et al. proposed a novel autoencoder framework of detecting pacing ECG. The results showed that the proposed method could achieve a significant performance with high accuracy, sensitivity, and F1-score through a series of experiments.

Recently, various deep learning techniques have been utilized to classify arrhythmias, including the use of one-dimensional convolutional neural network (CNN) model to handle the ECG signals in the time domain. Zhang et al. developed a new solution for cardiac arrhythmia classification in two dimensions by introducing the recurrence plot combined with an Inception-residual convolutional neural network-v2 (Inception-ResNet-v2). The results with only two leads of the 12-lead ECG original data showed that their proposed method achieved the highest average F1-score of 0.844, which outperformed other works. Jiang et al. proposed a hybrid attention-based deep learning network (HADLN) method for arrhythmia classification. The HADLN makes full use of the advantages of residual network and bidirectional long-short-term memory architecture to obtain fusion features containing local and global information and improve the interpretability of the model through the attention mechanism. Their experimental results showed that the HADLN method can achieve precision of 0.866, recall of 0.859, accuracy of 0.867, and F1-score of 0.880 on 10-fold cross-validation.

The clinical manifestations of myocardial ischemia (MI) are mainly the changes of ST-T segment of ECG. Nearly one third of patients with coronary heart disease, however, has no obvious ECG changes. Li et al. proposed a new method of detecting MI based on the T-wave area curve (TWAC). The preliminary test results showed that the sensitivity, specificity, and accuracy of the proposed method for detecting MI were 84.3, 83.6, and 84%, respectively, suggesting that the TWAC based approach may be an effective method for detecting MI, especially for the patients with no obvious ECG changes.

In summary, the 12 research papers in the Research Topic summarized the challenges and some of the most recent development and ideas in *Multi-Scale Computational Cardiology* and computational analysis of multimodal cardiovascular data, which will be useful for researchers working in the related fields.

## Author Contributions

LX wrote the manuscript. HZ and DZ revised the manuscript. All authors contributed to the article and approved the submitted version.

## Conflict of Interest

The authors declare that the research was conducted in the absence of any commercial or financial relationships that could be construed as a potential conflict of interest.

## Publisher's Note

All claims expressed in this article are solely those of the authors and do not necessarily represent those of their affiliated organizations, or those of the publisher, the editors and the reviewers. Any product that may be evaluated in this article, or claim that may be made by its manufacturer, is not guaranteed or endorsed by the publisher.

